# A summary of the current diagnostic methods for, and exploration of the value of microRNAs as biomarkers in, sepsis-associated encephalopathy

**DOI:** 10.3389/fnins.2023.1125888

**Published:** 2023-03-16

**Authors:** Zhang Yuechen, Xi Shaosong, Zhang Zhouxing, Gu Fuli, Hu Wei

**Affiliations:** Department of Critical Care Medicine, Affiliated Hangzhou First People’s Hospital, Zhejiang University School of Medicine, Hangzhou, China

**Keywords:** sepsis-associated encephalopathy, nervous system disease, microRNAs, sepsis, biomarker

## Abstract

Sepsis-associated encephalopathy (SAE) is an acute neurological deficit caused by severe sepsis without signs of direct brain infection, characterized by the systemic inflammation and disturbance of the blood–brain barrier. SAE is associated with a poor prognosis and high mortality in patients with sepsis. Survivors may exhibit long-term or permanent sequelae, including behavioral changes, cognitive impairment, and decreased quality of life. Early detection of SAE can help ameliorate long-term sequelae and reduce mortality. Half of the patients with sepsis suffer from SAE in the intensive care unit, but its physiopathological mechanism remains unknown. Therefore, the diagnosis of SAE remains a challenge. The current clinical diagnosis of SAE is a diagnosis of exclusion; this makes the process complex and time-consuming and delays early intervention by clinicians. Furthermore, the scoring scales and laboratory indicators involved have many problems, including insufficient specificity or sensitivity. Thus, a new biomarker with excellent sensitivity and specificity is urgently needed to guide the diagnosis of SAE. MicroRNAs have attracted attention as putative diagnostic and therapeutic targets for neurodegenerative diseases. They exist in various body fluids and are highly stable. Based on the outstanding performance of microRNAs as biomarkers for other neurodegenerative diseases, it is reasonable to infer that microRNAs will be excellent biomarkers for SAE. This review explores the current diagnostic methods for sepsis-associated encephalopathy (SAE). We also explore the role that microRNAs could play in SAE diagnosis and if they can be used to make the SAE diagnosis faster and more specific. We believe that our review makes a significant contribution to the literature because it summarizes some of the important diagnostic methods for SAE, highlighting their advantages and disadvantages in clinical use, and could benefit the field as it highlights the potential of miRNAs as SAE diagnostic markers.

## 1. Introduction

Sepsis is defined as life-threatening organ dysfunction caused by a dysregulated host response to infection (Singer et al., 2016). Sepsis is a critical healthcare problem, affecting tens of millions of people worldwide each year, with one in three to one in six deaths (Fleischmann et al., 2016; Reinhart et al., 2017; Rhee et al., 2017; Fleischmann-Struzek et al., 2020; Rudd et al., 2020). According to the National Institute of Health, sepsis-associated encephalopathy (SAE) is an acute diffuse neurological deficit caused by severe sepsis without signs of direct infection of the brain. It is characterized by systemic inflammation, disturbance of the blood-brain barrier (BBB), and changes in consciousness that can range from confusion to delirium or even lead to coma induction (Ebersoldt et al., 2007). SAE is one of the main manifestations of sepsis, which can manifest as the first organ dysfunction and contribute to a worse prognosis in patients with sepsis. The mortality rate increases with increasing severity (Gofton and Young, 2012) and affects up to 70% of patients with sepsis (Widmann and Heneka, 2014). Patients who survive SAE may exhibit long-term or permanent sequelae, including behavioral changes, cognitive impairment, decreased quality of life, or premature death (Feng et al., 2019). Data suggest that SAE occurs in 9 to 71% of cases (Sprung et al., 1990; Iacobone et al., 2009; Zhang et al., 2012; Molnár et al., 2018) and is one of the most common causes of encephalopathy in intensive care units (ICU) all over the world (Bleck et al., 1993; Eidelman et al., 1996; Ely et al., 2004). Differences in the incidence of SAE suggest that current diagnostic criteria are limited or not easily enforceable clinically. Given the enormous health impact of SAE, specific, accurate, and easy-to-use new diagnostic criteria for this condition are urgently needed.

## 2. Current diagnostic methods

**FIGURE 1:**
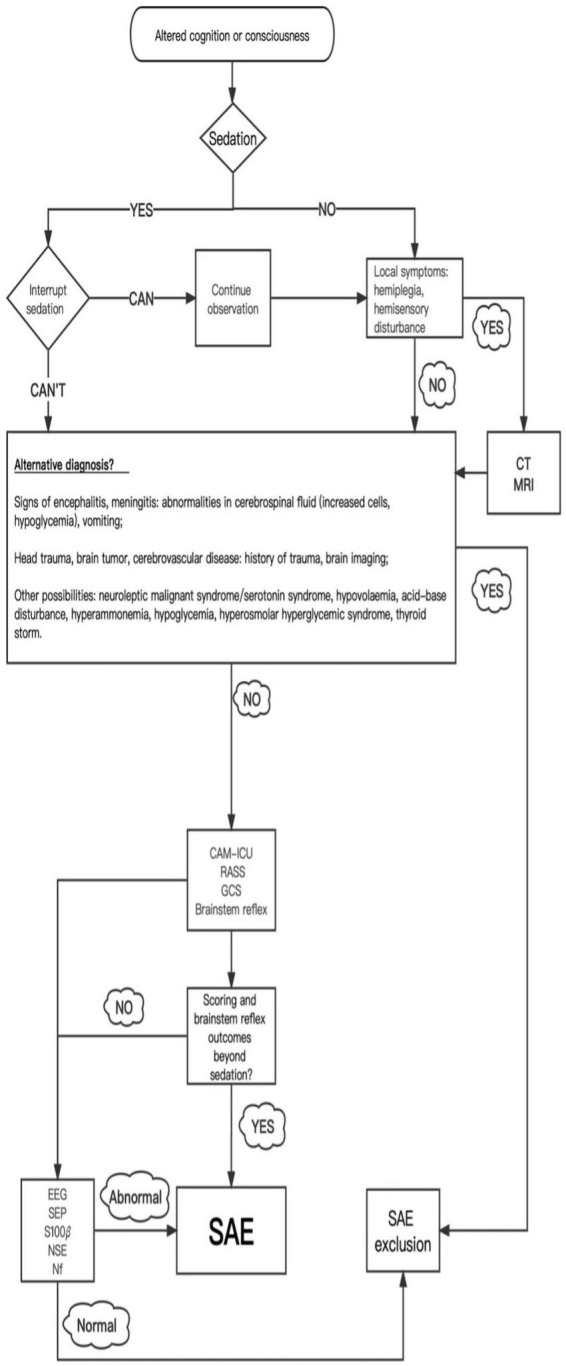
Diagnostic procedures for sepsis-associated encephalopathy.

Currently, SAE is still a diagnosis of exclusion. Based on the daily neurological assessment, when sepsis-related encephalopathy is suspected, it is first necessary to judge the patient’s mental status according to the rating scale and determine the manifestations of encephalopathy, which may be mild in the early stages. Secondly, an unabridged neurological examination is completed and necessary auxiliary tests are conducted to assist in the diagnosis or rule out other diagnoses. At the same time, it is essential to exclude the interference of sedatives, direct infection of the central nervous system, cerebrovascular disease, traumatic brain injury, brain tumors, metabolic encephalopathy, and drug side effects. It is also necessary to check the source of infection, obtain appropriate culture and drug sensitivity test results, actively treat sepsis, and give symptomatic and supportive treatment (Figure 1; Iacobone et al., 2009).

The rest of this paper describes the universal rating scales, auxiliary tests, and biomarkers used to diagnose SAE.

## 3. Rating scales

The general ward confusion assessment method (CAM) or the confusion assessment method in the intensive care unit (CAM-ICU) is used to assess a patient’s mental status. In their respective environments, multiple studies have demonstrated that CAM and CAM-ICU exhibit excellent sensitivity and specificity (CAM: 94–100%, 89–95%; CAM-ICU: 97–100%, 89–100%) (Inouye et al., 1990; Ely et al., 2001a,b; Vreeswijk et al., 2008; Wei et al., 2008; Toro et al., 2010). However, studies have shown that CAM is most likely to miss patients with quiet delirium (Khan et al., 2012), whereas CAM-ICU, although research has shown that it is a good ICU delirium screening scale, only has about 41–47% sensitivity when ICU nurses use it (van Eijk et al., 2011).

In ICU, sedatives are frequently used to improve the comfort and safety of patient, aid in the synchronization of patients with mechanical ventilation, and prevent accidental extubation (Devlin et al., 2001; Chen et al., 2022). However, the use of sedatives may have some side effects, such as excessive sedation leading to inability to awaken the patient even if the sedative is stopped or awakening accompanied by agitation, both of which will interfere with the judgment of the patient’s mental status (Ebersoldt et al., 2007; Van Rompaey et al., 2009; Lahariya et al., 2014; Junior et al., 2022). Therefore, before making a judgment, the sedation state should be evaluated through the Richmond Restlessness and Sedation Scale (RASS), which demonstrates excellent interrater reliability and criterion, construct, and face validity (Ely et al., 2003). A score of 0 means that the patient is in a conscious and natural state (Ely et al., 2003). It is generally recommended that a scale ≥ −1 be used for neurological examination or CAM-ICU evaluation (Ely et al., 2003; Van Rompaey et al., 2009; Lahariya et al., 2014; Junior et al., 2022).

The Glasgow Coma Scale (GCS) is a commonly used non-specific method to assess the degree of a coma and has been associated with the prognosis of sepsis-related encephalopathy (Sonneville et al., 2017). However, this study is based on the premise that GCS is one of the diagnostic criteria, and it cannot explain the advantages of GCS as a diagnostic method. The main limitations of GCS are that verbal responses are not assessable in mechanically ventilated patients and that brainstem examination is not directly considered (Sharshar et al., 2014).

The above scoring scales are non-specific methods and can therefore only be used for screening rather than diagnosis.

## 4. Neurological examination

Once the patient has altered cognition or consciousness, a detailed neurological examination is performed. This examination examines verbal stimuli, painful stimuli, brainstem reflexes, motor responses, and breathing patterns. Painful stimuli can elicit focal movements, withdrawal, postural reflexes, or unresponsiveness. Brainstem examination includes assessment of pupils and pupillary reactivity, spontaneous eye position and movements, vestibular-eyelid reflex, corneal reflexes, and cough and gag reflexes (Stevens and Bhardwaj, 2006; Sharshar et al., 2011). In deeply sedated patients, examination of pupil size, pupillary light reflex, corneal reflex, pain upon stimulation with a finger, ocular head response, and cough reflex may be more beneficial (Iacobone et al., 2009).

## 5. Metabolic analysis

Performing a comprehensive metabolic analysis includes a complete blood count and analyses of the blood glucose, blood gases, electrolytes, creatinine, ammonia, and alanine aminotransferase levels to look for other known causes of altered mental status, such as hypovolemia, hypoglycemia, diabetic ketoacidosis, uremic encephalopathy, hepatic encephalopathy, hypokalemia, hypoxemia, hypercapnia, and hyperemia (Iacobone et al., 2009; Mazeraud et al., 2020).

## 6. Electrophysiological examination

An electroencephalogram (EEG) is very sensitive in diagnosing sepsis-related encephalopathy. Young et al. (1992) studied 69 sepsis patients, 49 of whom had some degree of encephalopathy, classified as mild or severe. They identified five classes of gradually progressive EEG patterns: class 1 normal EEG, class 2: excessive θ rhythms, class 3: increased δ rhythm, class 4: triphasic, and class 5: suppression or burst suppression. Mortality in SAE is associated with the severity of EEG abnormalities: 0% for normal EEG, 19% for excessive θ rhythms, 36% for increased δ rhythm, 50% for triphasic waves, and 67% for suppression or burst suppression (Young et al., 1992). However, EEG is not highly specific, and EEG abnormalities are also common in other neurological injuries (Young et al., 1992; Hosokawa et al., 2014), and the use of sedatives can also lead to abnormal EEG signals (Young et al., 1992; Purdon et al., 2013; Akeju et al., 2014; Hosokawa et al., 2014).

Abnormal somatosensory evoked potentials (SEP) may also be beneficial electrophysiological markers of SAE (Zauner et al., 2002; Rosengarten et al., 2012; Hosokawa et al., 2014). Studies by Zauner et al. (2002) revealed that subcortical and cortical SEP pathways were impaired in 84 and 34% of all patients, respectively. In addition, subclinical cerebral focal signs were present in 24% of the subcortical SEP pathways and 6% of the cortical SEP pathways, but the results were not significantly different (Zauner et al., 2002). The advantage of SEP over EEG is that it is not affected by continuous sedation; however, the assessment of SEP is too cumbersome and may not be used routinely in the ICU (Zauner et al., 2002).

Despite more than 20 years of research, the role of electrophysiological examination in the diagnosis of SAE remains unclear, and most studies have significant limitations (Hosokawa et al., 2014).

## 7. Neuroimaging

Sharshar et al. (2007) showed that an “MRI of SAE included multiple ischemic strokes and white matter lesions at the level of the centrum semiovale, mainly surrounding Virchow–Robin spaces, ranging from small multiple areas to diffuse lesions, and characterized by hyperintensity on FLAIR images. The major lesions were also characterized by reduced signal on diffusion isotropic images and increased apparent diffusion coefficient” (Sharshar et al., 2007). Some patients with SAE exhibit vasogenic edema, which may suggest an increased BBB permeability or decreased brain autoregulation (Sharshar et al., 2007; Piazza et al., 2009; Polito et al., 2013).

A transcranial doppler ultrasound (TCD) has been used to measure cerebral blood flow velocity in SAE, where the impairment of cerebrovascular autoregulation can be founded, especially in the preclinical phase of SAE. During the first 2 days, most patients with severe sepsis have impaired cerebrovascular autoregulation (AR), and impaired AR is associated with SAE. In some cases, however, TCD did not find differences in cerebral perfusion (Schramm et al., 2012; Pierrakos et al., 2014).

Although neuroimaging in patients with SAE has a few defining characteristics and can be beneficial for diagnosis, its benefits have not been confirmed by multi-center clinical studies; therefore it is mostly used to rule out abscesses, tumors, trauma, and cerebrovascular diseases. Neuroimaging should be as perfect as possible in the case of focal neurological symptoms and changes in mental status.

## 8. Biomarkers

NT-proCNP, S100B, NSE, and NF are biomarkers of endothelial dysfunction, microglial activation, and brain injury with concomitant axonal damage. Although their enhancements help diagnose SAE (Yao et al., 2014; Ehler et al., 2017; Ehler et al., 2019a,b; Khan et al., 2020), their sensitivity and specificity are not enough. Moreover they have not been clinically validated by multi centers, which limits their clinical use (Table 1).

**TABLE 1:** The presentation of several biomarkers of sepsis-associated encephalopathy.

Biomarkers	Sensitivity and specificity
NT-proCNP	NA, *p* < 0.01 (Ehler et al., 2019b)
S-100B	85.4% sensitivity, 67.2% specificity (Yao et al., 2014)
NSE	54.2% sensitivity, 82.8% specificity (Yao et al., 2014)
NF	NA (*R* = 0.53, *p* = 0.045) (Ehler et al., 2017); NA (*R* = 0.534, *p* = 0.022) (Ehler et al., 2019a)

NT-proCNP, C-type natriuretic peptide; S-100B, S100 calcium-binding protein beta subunit; NSE, neuron-specific enolase; NF, neurofilament.

## 9. Diagnostic value of microRNAs in SAE

MicroRNAs non-coding RNAs that are about 22-nt long; they induce mRNA degradation or repress protein translation by binding to target messenger RNAs (mRNAs), thus playing a role in gene silencing (Bartel, 2009; Jonas and Izaurralde, 2015; O’Brien et al., 2018). microRNAs play significant roles in various neurobiological processes, such as differentiation, cell proliferation, metabolism, cell cycle regulation, and apoptosis (Nowakowski et al., 2018).

The high degree of conservation of microRNAs determines that a single miRNA can target multiple genes and regulate different biological pathways (Valinezhad Orang et al., 2014). Under pathological conditions, such as neurodegenerative diseases, network-based patterns of gene regulation converge on distinct biological pathways to drive disease phenotypes (Salta et al., 2016; Wilk and Braun, 2018; Juźwik et al., 2019).

In addition, microRNAs have other advantages as biomarkers. For example, they are present in various bodily fluids, including blood, urine, and saliva, thus allowing relatively non-invasive sample collection (Ciesla et al., 2011). In addition to accessibility, microRNAs are highly stable in various body fluids and different biological specimens and can be efficiently detected and amplified by means of molecular biology tools such as real-time PCR or small RNA sequencing (Mitchell et al., 2008; Ciesla et al., 2011; Enuka et al., 2016; Beltrán-García et al., 2020). Moreover, current evidence suggests that the BBB does not prevent the passage of microRNAs between CSF and blood (Stoicea et al., 2016). microRNAs can enter the blood from brain tissue through the BBB under pathological conditions, making them potential biomarkers for central nervous system diseases (Dalkara and Alarcon-Martinez, 2015).

Previous studies have shown that microRNAs, principal gene regulators in different biological pathways, are closely related to disease progression and play crucial roles in the initiation and progression of neurodegenerative diseases as well as in neuronal differentiation and synaptic plasticity (Lehmann et al., 2012; Pasquinelli, 2012; Sohel, 2016; Nowakowski et al., 2018). microRNAs, have been widely studied and can be used in the diagnosis of Alzheimer’s Disease, Parkinson’s Disease, multiple sclerosis, Huntington’s Disease, epilepsy, and other diseases (Junker et al., 2011; Henshall et al., 2016; Miniarikova et al., 2018; Sadlon et al., 2019; Doxakis, 2020; Singh and Yadav, 2020; Bazrgar et al., 2021). Therefore, we infer that microRNAs have the potential to be SAE biomarkers (Table 2).

**TABLE 2:** Systematic table of all miRNAs and their expression levels, their correlations with SAE progression, and the mechanisms underlying their action.

miRNAs	Expression level	Correlation	Mechanism involved
miR-370-3p	Middle to high	Positive	SUMOylation pathways: Post-translational modification processes of several cellular mechanisms including apoptosis. Overexpression of miR-370-3p enhanced sensitivity to apoptosis upon activation by cytokines or LPS (Visitchanakun et al., 2020).
miR-130a-3p	High Low	Positive Negative	Overexpression of miR-130a-3p abolished the role of YY1 in promoting microglial M2 polarization (Peng et al., 2022). Downregulation of miR-130a leads to the upregulation of the expression of IL-18, which aggravates thrombocytopenia in severe sepsis (Cui et al., 2016).
miR-25-3p	Low	Negative	miR-25-3p inhibits the NLRP3/IL-1β/IL-18 axis by directly targeting TLR4, thereby attenuating microglia activation in SAE (Yao et al., 2015; Antonakos et al., 2022; Luo et al., 2022).
miR-9-5p	Low	Negative	miR-9-5p alleviates sepsis-induced ferroptosis by inhibiting the expression of TFRC and GOT1 *in vivo* (Wei et al., 2022).
miR-155	High*	Positive*	miR-155 reduces the expression of SOCS1 in microglia, and SOCS1 is a key inhibitor of inflammatory response (Guo et al., 2019).
miR-27a	Low*	Negative*	miRNA-27a negatively modulates the inflammatory response in lipopolysaccharide-stimulated microglia by targeting TLR4 and IRAK4 (Lv et al., 2017).
miR-210	High*	Positive*	miR-210 induces microglial M1 activation by targeting SIRT1, thereby reducing deacetylation of NF-κB subunit p65 and increasing NF-κB signaling activity (Li et al., 2020).

*What we expected.

Research shows that miR-370-3p levels increase in the brain and plasma in SAE, but not in uremic encephalopathy. Overexpression of miR-370-3p enhances sensitivity to apoptosis upon activation by cytokines or LPS. TNF-α induces apoptosis of PC-12 neurons by activating the expression of miR-370-3p in PC-12 neurons (Visitchanakun et al., 2020). miR-130a-3p is upregulated in SAE. YY1 can promote microglial M2 polarization by inhibiting miR-130a-3p promoter activity, thereby improving SAE (Peng et al., 2022). miR-25-3p is expressed at low levels in the cerebral cortex of SAE mice, while microglia are overactivated. *In vitro* experiments demonstrated that increasing miR-25-3p expression can attenuate microglial activation in SAE by directly targeting TLR4 (toll-like receptor 4) to inhibit the NLRP3/IL-1β/IL-18 axis, suggesting that miR-25-3p plays a protective role against SAE (Luo et al., 2022). Sepsis induced high expression of serous exosome-derived NEAT1, which might exacerbate SAE by regulating the miR-9-5p/TFRC and GOT1 axis to promote siderosis (Wei et al., 2022). Although there are few microRNAs studied in SAE, it is expected that some microRNAs in sepsis may be related to SAE, especially some microRNAs related to inflammation.

In severe sepsis with thrombocytopenia, miR-130a is downregulated. The mechanism is that the downregulation of miR-130a leads to upregulation of the expression of IL-18t, which aggravates thrombocytopenia in severe sepsis (Cui et al., 2016). Notably, this is contrary to what Peng et al. (2022) observed in SAE, arguably both SAE and severe sepsis with thrombocytopenia are a more severe subtype of sepsis, and the expression of miR-130a should be consistent, which deserves further study by future researchers. In the study by Yao et al. (2015) miR-25 was the most significantly altered RNA in sepsis, and the decrease of miR-25 levels correlated with the severity of sepsis, SOFA score, CRP, and PCT levels, which is similar to the results observed by Luo et al. (2022), Antonakos et al. (2022). Dysregulation of circulating miR-25 has also been described in other inflammatory settings such as periodontitis and vascular endothelial cell inflammation, further highlighting the potential of miR-25 as a biomarker.

Microglia are generally considered as macrophages in the central nervous system. After activation, microglia can cause neuronal damage and even apoptosis by releasing inflammatory mediators, reactive oxygen species, neurotransmitters, and other substances. Therefore, some microRNAs that play a central role in microglia are also expected to affect the progression of SAE. miR-155, miR-27a, and miR-210 are widely recognized as biomarkers in sepsis, and recent studies have shown that they also play a central role in microglial function (Huang et al., 2014; Szilágyi et al., 2019; Osca-Verdegal et al., 2021).

miR-155 mediates LPS-induced neuroinflammation by modulating microglia, and inhibition of miR-155 contributes to the establishment of endotoxin tolerance. The mechanism is that inhibition of miR-155 enhances the expression of SOCS1 in microglia, and SOCS1 is a key inhibitor of inflammatory response (Guo et al., 2019). Therefore, it is expected that high levels of miR-155 will be found in SAE.

miR-27a levels rapidly decrease in microglia after LPS stimulation, and overexpression of miR-27a significantly reduces the production of inflammatory cytokines, such as IL-6, IL-1β, TNF-α, and NO. miR-27a directly inhibits the expression of TLR4 and IRAK4—key adapter kinases in the TLR4/MyD88 signaling pathway (Lv et al., 2017).

miR-210 inhibitor can effectively suppress microglia-mediated neuroinflammation and significantly reduce HIE-induced brain damage. miR-210 induces microglial M1 activation by targeting SIRT1, thereby reducing deacetylation of NF-κB subunit p65 and increasing NF-κB signaling activity (Li et al., 2020). Low levels of miR-210 exhibit neuroprotective effects in mice with hypoxic-ischemic encephalopathy (Ma et al., 2016; Li et al., 2020); its effect in SAE, however, is yet to be studies.

Although, in theory, microRNAs are excellent biomarkers, they have their disadvantages. A prominent issue is how microRNAs recognize partially complementary sequences. Smaller microRNAs provide a limited amount of sequence information for specificity. Furthermore, since partial pairing between microRNAs and target sites is usually sufficient, the range of genes that can be regulated is relatively broad. This implies that a single miRNA can regulate multiple mRNAs, and predicting these targets is not easy (Bartel, 2009). However, given the challenges of matching microRNAs to specific target sequences, several methods have been developed to identify miRNA targets, ranging from small-scale genetic studies to biochemical approaches such as algorithmic prediction and high-throughput sequencing to isolate target mRNAs or sequences (Thomson et al., 2011).

## 10. Conclusion

In conclusion, sepsis-related encephalopathy is a complex central nervous system injury causing long-term sequelae in patients, resulting in a poor prognosis and lower quality of life. SAE is still diagnosed by exclusion. Some biomarkers have been used to assess brain dysfunction caused by sepsis, but the evidence is insufficient, resulting in invalid clinical measurements.

As chief gene regulators, microRNAs are closely related to disease progression. Because of their stable structure, presence in various body fluids, and ability to pass through the BBB, they are theoretically excellent biomarkers for diagnosis and prognosis of central nervous system diseases. However, they have some disadvantages. Due to the wide range of downstream targets of microRNAs, a single miRNA can interfere with the immune response in multiple ways and simultaneously manifest the multi-organ damage of sepsis; therefore, some differential microRNAs may not be attributed to SAE. If they are used for diagnosis, they will lead to misdiagnosis, and any treatment guided by them will bring immeasurable harm to patients. The complexity of miRNA interactions underscores the challenge of having to take a holistic view of the impact of miRNAs on the immune response. Thus, we must be rigorous and cautious when identifying biomarkers, especially with potential molecules with a wide range of targets. Additionally, the combined use of multiple miRNAs and/or other biomarkers associated with sepsis may be an effective method for diagnosing SAE because the miRNA expression profile is not SAE-specific. Undoubtedly, the postulation of microRNAs as biomarkers of SAE requires more research in the future, but it will be worthwhile.

## Author contributions

ZY, XS, and HW contributed to the conception of the review and revised the manuscript. ZY was in charge of collating the literature and wrote the first draft of the manuscript. ZY, XS, ZZ, and GF wrote the sections of the manuscript. All authors contributed to the manuscript and approved the submitted version.
